# *Lactococcus lactis* subsp. *cremoris* C60 Upregulates Macrophage Function by Modifying Metabolic Preference in Enhanced Anti-Tumor Immunity

**DOI:** 10.3390/cancers16101928

**Published:** 2024-05-18

**Authors:** Suguru Saito, Duo-Yao Cao, Toshio Maekawa, Noriko M. Tsuji, Alato Okuno

**Affiliations:** 1 Department of Infection and Immunity, Division of Virology, Faculty of Medicine, Jichi Medical University, Shimotsuke, Tochigi 3290431, Japan; 2 Department of Biomedical Sciences, Cedars-Sinai Medical Center, Los Angeles, CA 90048, USA; duoyao.cao@cshs.org; 3 iFoodMed Inc., Tsuchiura, Ibaraki 3000873, Japan; maekawa@ifoodmed.jp; 4 Department of Pathology and Microbiology, Division of Immune Homeostasis, Nihon University School of Medicine, Itabashi, Tokyo 1738610, Japan; tsuji.noriko@nihon-u.ac.jp; 5 Department of Pathology and Microbiology, Division of Microbiology, Nihon University School of Medicine, Itabashi, Tokyo 1738610, Japan; 6 Department of Food Science, Jumonji University, Niiza, Saitama 3528510, Japan; 7 Department of Health and Nutrition, Faculty of Human Design, Shibata Gakuen University, Hirosaki, Aomori 0368530, Japan

**Keywords:** probiotics, macrophage, anti-tumor immunity, glycolysis, ATP

## Abstract

**Simple Summary:**

*Lactococcus lactis* subsp*. cremoris* C60, a probiotic lactic acid bacteria (LAB) strain, induces immunomodulation in myeloid cells, including macrophages. C60 was reported to suppress murine melanoma in our previous study, while the effect of this strain on macrophage anti-tumor activity is still uncharacterized. Here, we report that C60 enhances macrophage function by altering metabolic preference to glycolysis, allowing the cell to produce more adenosine triphosphate (ATP) and maintain an inflammatory status. Intratumor (IT) macrophages in C60-administered mice increase glycolysis rather than fatty acid oxidation (FAO) in their metabolism, which enables the cells to generate sufficient energy resources to increase their immune activity, including antigen-dependent CD8+ T cell activation in a murine melanoma model. This is the first report demonstrating that probiotic LAB assists macrophage function in anti-tumor immunity, providing a novel possibility for LAB adaptation in maintaining our health.

**Abstract:**

*Lactococcus lactis* subsp. *cremoris* C60 is a probiotic strain of lactic acid bacteria (LAB) which induces various immune modifications in myeloid lineage cells. These modifications subsequently regulate T cell function, resulting in enhanced immunity both locally and systemically. Here, we report that C60 suppresses tumor growth by enhancing macrophage function via metabolic alterations, thereby increasing adenosine triphosphate (ATP) production in a murine melanoma model. Intragastric (i.g.) administration of C60 significantly reduced tumor volume compared to saline administration in mice. The anti-tumor function of intratumor (IT) macrophage was upregulated in mice administered with C60, as evidenced by an increased inflammatory phenotype (M1) rather than an anti-inflammatory/reparative (M2) phenotype, along with enhanced antigen-presenting ability, resulting in increased tumor antigen-specific CD8+ T cells. Through this functional modification, we identified that C60 establishes a glycolysis-dominant metabolism, rather than fatty acid oxidation (FAO), in IT macrophages, leading to increased intracellular ATP levels. To address the question of why orally supplemented C60 exhibits functions in distal places, we found a possibility that bacterial cell wall components, which could be distributed throughout the body from the gut, may induce stimulatory signals in peripheral macrophages via Toll-like receptors (TLRs) signaling activation. Thus, C60 strengthens macrophage anti-tumor immunity by promoting a predominant metabolic shift towards glycolysis upon TLR-mediated stimulation, thereby increasing substantial energy production.

## 1. Introduction

Probiotics utilizing lactic acid bacteria (LAB) are a well-known approach that maintains and strengthens host immunity [1,2]. While the immunomodulatory effects of probiotic LAB are diverse, the core mechanism is based on functional enhancement of myeloid lineage cells, such as dendritic cells (DCs) and macrophages in most cases [3,4,5]. Due to their nature, these myeloid cells have a strong capability to capture exogenous bacteria, even though they are non-pathogenic, through extracellular pattern recognition receptors (PRRs), then process them in the cytosol [6]. Both these extracellular contact and intracellular processed mechanisms generate stimulatory signals in myeloid cells via bacteria-originated substances represented by cell wall components, nucleic acids, and others [6,7]. This mechanistic process modifies the cellular function of the myeloid cells, which eventually strengthens the immune response of the cells [8,9,10]. Most former studies have revealed that LAB signals trigger cellular activation and cytokine production in myeloid cells [6,11]. These functional modifications eventually enhance antigen presenting activity, directly activating T cells as an innate–adaptive immunity axis in LAB probiotics [12,13]. 

*Lactococcus lactis* subsp. *cremoris* C60 is a probiotic LAB strain that was characterized as inducing T cell activation via enhancing DC and macrophage activation in our previous studies [14,15]. Similar to other LAB strains, our initial studies revealed that C60 stimulation increased pro-inflammatory cytokine production and cellular activation in DCs and macrophages, resulting in enhanced antigen presentation to T cells. This effect maintained immunological homeostasis and protective environment in the intestine, which was obviously observed in aged mice. In general understanding, the direct effect of orally administered probiotic LAB is initiated in the gut, while some observations have proven that the effect can be extended to distal tissues and organs from the gut—for instance, LAB administration attenuated asthma and airway hypersensitivity in mice [16,17]. Moreover, LAB suppressed food allergy in a mouse model [18]. We sought other possibilities in the attenuation/prevention of specific diseases by adapting C60, and we eventually found that C60 administration suppressed tumor growth in a B16-ovalbumin (OVA)-originated murine melanoma model [19]. We revealed that C60 enhanced major histocompatibility complex (MHC) class I-dependent antigen presentation machinery by activating 20S immunoproteasome, which consequently increased the differentiation of tumor antigen-specific CD8^+^ T cells as major players in tumor killing. This is the first finding to provide solid evidence that the immunomodulatory function of probiotic LAB is able to enhance anti-tumor immunity directly. However, we still need to provide answers as to whether other immune cells are functionally enhanced by C60 in the cancer environment. Especially for macrophages, composing a predominant population in the tumor infiltrating leukocytes in the melanoma tumor microenvironment (TME), they may obtain functional modification by C60, which augments anti-tumor immunity. With this background, we decided to investigate how C60 affects macrophage function in a murine melanoma model.

## 2. Materials and Methods

### 2.1. Lactic Acid Bacteria Culture

*Lactococcus lactis* subsp. *cremoris* C60 was cultured in MRS broth (BD Difco^TM^, BD Bioscence, Franklin Lakes, NJ, USA) at 30 °C for 24 h. The bacterial colony forming unit (CFU/mL) was calculated in each culture. For HK-C60 preparation, the bacteria were autoclaved at 95 °C for 10 min, then the bacterial cells were precipitated by centrifugation at 5000× *g* for 10 min. After being washed with saline (0.9% NaCl), the pellet was finally resuspended in saline (for in vivo administration) or phosphate buffered saline (PBS; for in vitro culture). The suspension was used as HK-C60 for each experiment. The samples were stored at −80 °C until use and avoided freeze and thaw. 

### 2.2. Isolation of Bacterial Cell Wall Extract

Cell wall extract was isolated from C60 following a method described in a previous publication [20]. The C60 (5.0 × 10^9^ CFU/mL) suspension was centrifuged at 5000× *g* for 10 min to harvest the bacteria cells and washed with PBS before the cell pellet was resuspended in PBS. The suspension was stored at −80 °C overnight, then thawed at room temperature (RT). The bacterial cells were crushed with 0.3 mm stainless beads followed by sonication at 4 °C for 20 min. The cell wall extract (CWE) was precipitated by centrifugation at 5000× *g* for 10 min and washed with PBS. The sample was autoclaved at 95 °C for 10 min, then the CWE was collected by centrifugation at 5000× *g* for 10 min. Finally, the pellet was resuspended in PBS and stored at −80 °C until use and avoided freeze and thaw. For fragmented CWE preparation, the CWE was further sonicated at 4 °C overnight, then centrifuged at 5000× *g* for 10 min to confirm that all structures in the CWE were sufficiently fragmented allowing them to exist in the supernatant as small molecules. 

### 2.3. Cell Culture 

B16-Ovalbumin (OVA) expressing cells were obtained from the American Type Culture Collection (Manassas, VA, USA). The frozen stock was thawed on ice and cultured in Dulbecco’s Modified Eagle Medium (DMEM) high-glucose medium (4.5 g/L of glucose) supplemented with 10% Fetal Bovine Serum (FBS), 100 mg/mL penicillin, and 100 mg/mL streptomycin. The cells were pre-cultured for at least two generations before inoculation into mice.

### 2.4. Mice and Tumor Model

C57BL/6 mice were purchased from CLEA Japan (Tokyo, Japan) and The Jackson Laboratory (Bar Harbor, ME, USA). OT-I transgenic mice (C57BL/6-Tg (TcraTcrb) 1100Mjb/J), TLR2-KO mice (B6.129-Tlr2tm1Kir/J), and TLR4-KO mice (B6(Cg)-Tlr4tm1.2Karp/J) were also obtained from The Jackson Laboratory. All mice were bred in the same facility, at least for 8 weeks to keep the same intestinal environment before using for experiments. The mice were maintained in a specific pathogen-free (SPF) facility with 12-h light/dark cycles and allowed free access to food and water. Adult mice of both genders aged 8 to 20 weeks were used for each experiment. Saline (100 μL) or HK-C60 (100 μL of HK-C60 suspension (5.0 × 10^9^ CFU/mL in saline)) was administered to the mice intragastrically (i.g.) using a disposable plastic needle every day for 28 days. The dose of HK-60 was decided by following the result of pre-screening (Figure S1). After 14 days of oral administration, the mice received tumor inoculation (1.0 × 10^6^ of B16-OVA cells in 100 μL of PBS) on the skin of the back by subcutaneous (s.c.) injection. Tumor volume was measured at day 7 and day 14 after tumor inoculation in each mouse. Tumor volumes were calculated using the formula: Volume (V) = (Length (L) × Width (W)^2^) × 0.52 (L > W). At day 28 of oral administration, the mice were sacrificed and used for analysis. All animal experimental protocols were approved by the animal care and use committee of Jichi Medical University (20038-01) and Shibata Gakuen University (2107).

### 2.5. Flow Cytometry

Flow cytometry analysis was performed using LSRII (BD Biosciences, Franklin Lakes, NJ, USA) and FACS Aria II (BD Biosciences). For extracellular marker staining, the samples were incubated with fluorochrome-conjugated monoclonal antibodies (mAb) or tetramer in the presence of anti-CD16/CD32 mAb for blocking of Fc gamma Receptor (FcγR) II/III at 4 °C for 30 min. Intracellular staining was performed by using the BD Cytofix/Cytoperm™ Fixation/Permeabilization Kit (BD Biosciences). For intracellular staining, the extracellular-stained cells were fixed at 4 °C for 20 min followed by staining of intracell-lular targets at 4 °C for 30 min. For CD8+ T cell intracellular cytokine detection, the isolated or cultured cells were restimulated with phorbol 12-myristate 13-acetate (PMA, 100 ng/mL) (Sigma-Aldrich, Burlington, MA, USA) and ionomycin (250 ng/mL) (Sigma-Aldrich) in the presence of GolgiStop^TM^ (1 μg/mL) (BD Biosciences) at 37 °C for 5 h, followed by the staining procedure. In the reactive oxygen species (ROS) production assay, the cells were stained with 2′,7′-dichlorodihydrofluorescein diacetate (H2DCFDA) (5 μM) (Thermo Fisher Scientific, Waltham, MA, USA) at 37 °C for 60 min. Mitochondrial staining was performed by using tetramethylrhodamine, ethyl ester (TMRE) (100 nM) (Thermo Fisher Scientific) or MitoTrackerTM (500 nM) (Thermo Fisher Scientific) at 37 °C for 60 min, respectively. The antibodies and tetramer used in flow cytometry analysis are represented in Supplemental Table S1. The data were analyzed by using FlowJo 10 (BD Biosciences).

### 2.6. Real-Time Polymerase Chain Reaction (Real-Time PCR)

The total RNA was isolated from macrophages using TRIzol^®^ reagent (Thermo Fisher Scientific). The concentration and purity of RNA were measured by using NanoDrop 2000c (Thermo Fisher Scientific). Samples with A_260_/A_280_ ratios between 1.9 to 2.0 were selected for subsequent procedures. Total RNA (250–500 ng) was used for reverse transcription to generate complementary DNA (cDNA) using the PrimeScript™ RT-PCR Kit (Takara, Tokyo, Japan). The cDNA was used for quantitative PCR, which was performed using a Ther-mal Cycler Dice^®^ Real Time System III (Takara). mRNA expressions were quantified using the ΔCt method. The primer sequences used in the assay are listed in Supplemental Table S2.

### 2.7. Tumor Cell Isolation

Tumor infiltrating immune cells were isolated from tumor following a method described in a previous publication [21]. The tumor was excised from the mouse and briefly washed with PBS, then chopped by scissors and crushed on a 70 μm cell strainer. The sample was digested with collagenase (1 mg/mL) at 37 °C for 15 min, then refiltered through a 70 μm cell strainer followed by washing with Roswell Park Memorial Institute (RPMI) 1640 complete medium (supplemented with 10% FBS, 100 µg/mL penicillin, 100 µg/mL streptomycin). Tumor in-filtrating leukocytes were isolated from the tumor cells by percoll gradient centrifugation at 600× *g* for 20 min. The intermediate cell layer was collected as tumor infiltrated leukocytes. Macrophages were then isolated from the leukocytes using the EasySep™ Mouse F4/80 Positive Selection Kit (Stemcell Technologies, Vancouver, BC, Canada). The purity of macrophages was assessed by flow cytometry, and samples with more than 90% of CD11b+F4/80+ population were used for experiments.

### 2.8. Tumor Killing Assay

IT macrophages (Effector; 1.0 × 10^6^/mL) and B16-OVA cells (Target; 1.0 × 10^6^/mL) were mixed at a 1:1 ratio in RPMI complete medium, then cultured at 37 °C for 16 h. Some cultures were prepared with only IT macrophages or B16-OVA cells for controls. The cultured medium (CM) was harvested and stored at −80 °C until use. The cytotoxicity of IT macrophages against tumor cells was measured by assessing lactate dehydrogenase (LDH) release from B16-OVA cells in CM. The released LDH was measured by absorbance at 490 nm (background) and 680 nm (target) using the Pierce LDH Cytotoxicity Assay Kit (Thermo Fisher Scientific). Cytotoxicity was calculated using the formula represented in the kit manual: % Cytotoxicity = (Co-culture’s LDH activity − Spontaneous Target LDH activity)/(Maximum Target LDH activity − Spontaneous Target LDH activity) × 100. All procedures were performed by following the product manual. 

### 2.9. Antigen Uptake Assay

IT macrophages (1.0 × 10^6^/mL) were incubated with FITC-labeled OVA (OVA-FITC; 500 ng/mL) (Thermo Fisher Scientific) in RPMI complete medium at 37 °C for 2 h. The cells were then washed with PBS containing 2% FBS (PBS/2% FBS) and analyzed by flow cy-tometry. The fluorescence signal (mean fluorescence intensity; MFI) originating from intracellularly captured OVA-FITC was used to assess the antigen uptake ability in macrophages.

### 2.10. Antigen Presentation Assay

CD8+ T cells were isolated from the spleen of OT-I mice using the CD8a+ T Cell Isolation Kit, mouse (Miltenyi Biotec, Bergisch Gladbach, North Rhine-Westphalia, Germany). All isolation procedures were performed by following the manufacturer’s instructions. The purity of CD8+ T cells was assessed by flow cytometry, and samples with more than 90% of CD3+CD8+ population were used for experiments. The OT-I CD8+ cells (5.0 × 10^6^/mL) and IT macrophages (1.0 × 10^6^/mL) were co-cultured in the presence of OVA (100 μg/mL) at 37 °C for 24 h. The CD69 expression and TNF-α production in CD8+ T cells were analyzed by flow cytometry.

### 2.11. Adenosine Triphosphate (ATP) Assay

The IT macrophages were washed with PBS, then resuspended in PBS at 2.5 × 10^6^/mL. A 100 μL aliquot of the cell suspension (2.5 × 10^5^ cells) was mixed with an equal volume of CellTiter-Glo^®^ 2.0 reagent and incubated at room temperature for 15 min. Luminescence intensity was measured by using a microplate reader. In some assays, the IT macrophages (1.0 × 10^6^/mL) were cultured in RPMI1640 complete medium (1 g/L of glucose) or RPMI1640 high glucose (4.5 g/L of glucose, supplemented with 10% FBS (fatty acid de-pleted), 100 µg/mL penicillin, 100 µg/mL streptomycin) at 37 °C for 6 h followed by ATP measurement.

### 2.12. Glucose Uptake Assay

IT macrophages (1.0 × 10^6^/mL) were incubated with the glucose analog 2-(7-nitro-2,1,3-benzoxadiazol-4-yl)-D-glucosamine (2-NBDG; 100 μM, Thermo Fisher Scientific) at 37 °C for 60 min. In some assays, the IT macrophages were first pre-cultured with vehicle (PBS), HK-C60 (5.0 × 10^7^ CFU/mL), or CWE (isolated from 5.0 × 10^7^ CFU/mL) at 37 °C for 60 min, then subjected to a glucose uptake assay. Moreover, some cultures were pre-treated with vehicle (DMSO), TL2-C29 (TLR2 inhibitor, 10 μM; InvivoGen, San Diego, CA, USA), or Sparstolonin B (SsnB, TLR4 inhibitor, 100 μM; Sigma-Aldrich) at 37 °C for 60 min, then subsequently subjected to a glucose uptake assay in the presence of HK-C60 (5.0 × 10^7^ CFU/mL) or CWE (isolated from 5.0 × 10^7^ CFU/mL) at 37 °C for 60 min. The mean fluorescence intensity (MFI) of uptaken 2-NBDG was measured by flow cytometry.

### 2.13. Lipid Uptake Assay

IT macrophages (1.0 × 10^6^/mL) were incubated with oleic acid (OA; 200 μM) in RPMI1640 medium supplemented with 1% bovine serum albumin (BSA) at 37 °C for 6 h, then the intracellular lipid droplets were stained with Lipi Deep Red (LDR, 200 μM; Dojin Chemical, Tokyo, Japan) at 37 °C for 60 min. The MFI of LDR was measured by flow cytometry.

### 2.14. Cytokine Production Assay

IT macrophages (1.0 × 10^6^/mL) were incubated with vehicle (PBS), HK-C60 (5.0 × 10^7^ CFU/mL), or CWE (isolated from 5.0 × 10^7^ CFU/mL) at 37 °C for 6 h. Some cultures were pre-treated with vehicle (DMSO), TL2-C29 (10 μM), or SsnB (100 μM) at 37 °C for 60 min, then subsequently cultured in the presence of HK-C60 (5.0 × 10^7^ CFU/mL) or CWE (isolated from 5.0 × 10^7^ CFU/mL) at 37 °C for 6 h. Intracellular TNF-α was stained and analyzed by flow cytometry.

### 2.15. Real-Time Metabolic Assay

IT macrophages (1.5 × 10^5^/100 μL) were seeded on Cell-Tak (Corning, Corning, NY, USA) coated Seahorse XF96 plates (Agilent Technologies, Santa Clara, CA, USA) in RPMI 1640 medium. Analysis was performed using oligomycin (2 μM), FCCP (carbonyl cyanide-p-trifluoromethoxyphenylhydrazone, 500 nM), and rotenone (200 nM) with antimycin A (1 μM) added to the wells, and the respiratory oxygen consumption rate was measured by the Seahorse XF96 analyzer (Agilent Technologies). Metabolic parameters were calculated following the method described in a previous report [22].

### 2.16. Metabolomics

IT macrophages were washed with saline, and the cell pellets were frozen at −80 °C until subsequent treatment. For metabolite extraction, the cell pellets were treated with extraction buffer (80% methanol containing 0.85% ammonium bicarbonate). The samples were vortexed for 30 s, then stored at −80 °C for 30 min or longer followed by centrifugation at 15,000× *g* for 15 min at 4 °C; then, supernatants were collected as metabolite containing fractions. The organic solvent was evaporated by speedvac and dried samples were resuspended in 20% methanol. Metabolite extracts were analyzed by LC-MS following the system described in a previous publication [23]. Briefly, the samples were analyzed by an LC-MS system which was an Agilent 1290 LC linked to a Brukler Impact II QTOF Mass spectrometer. The samples were injected into an Agilent Eclipse Plus reversed phase C18 column (2.1 mm × 150 mm, 1.8 μm particle size, 95 Å pore size) for separation. Solvent A was 0.1% (*v*/*v*) formic acid in water, and solvent B was 0.1% (*v*/*v*) formic acid in acetonitrile. The chromatographic conditions were as follows: t = 0 min, 25% B; t = 10 min, 99% B; t = 15 min, 99% B; t = 15.1 min, 25% B; t = 18 min, 25% B. The flow rate was 400 μL/min. All MS spectra were obtained in the positive ion mode. The MS conditions used for Q-TOF were as follows: nebulizer, 1.0 bar; dry temperature, 230 °C; dry gas, 8 L/min; capillary voltage, 4500 V; end plate offset, 500 V; spectra rate, 1.0 Hz. The raw data were exported by Bruker Data Analysis 4.5. as.csv files, and the.csv files were processed by IsoMS Pro 1.2.15. The data files were uploaded to Metaboanalyst 6.0 (www.metaboanalyst.ca, accessed on 14 May 2024) for analysis.

### 2.17. Statistics

Statistical analyses were performed using GraphPad Prism 10.0 (GraphPad Software, San Diego, CA, USA). Student *t*-test and one-way analysis of variance (ANOVA) were used to analyze the data for significant differences.

## 3. Results

### 3.1. C60 Suppresses Tumor Growth by Inducing a Predominantly Inflammatory Phenotype in Macrophages

**Figure 1:**
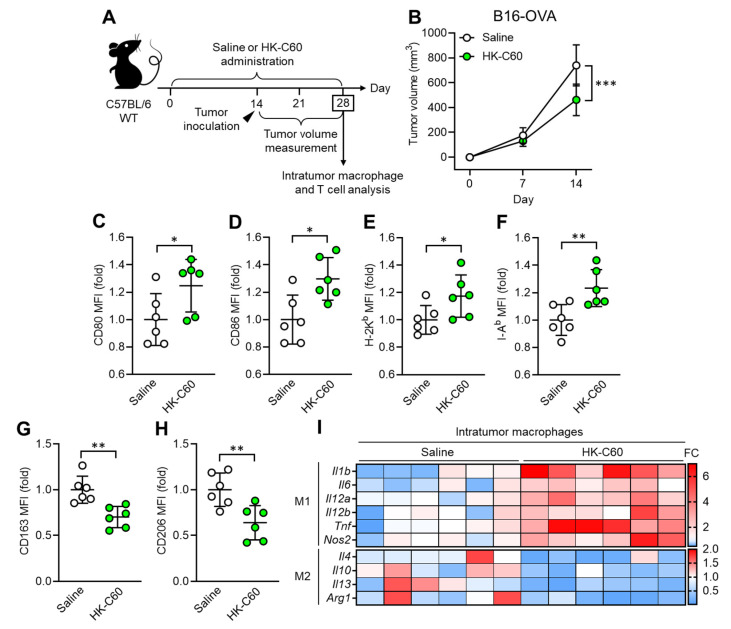
C60 administration suppresses tumor growth accompanied with macrophage polarization into inflammatory phenotype. (**A**) Experimental design of intragastric (i.g.) administration and murine tumor model. WT mice received i.g. administration of saline or HK-C60 for 14 days, then B16-OVA cells were inoculated into the mice by s.c. injection. The tumor volumes were measured after 7 and 14 days of post tumor inoculation (in day 21 and day 28 in the administration schedule). In the last day (day 28), the mice were sacrificed, and tumor isolated leukocytes were subjected to macrophage and T cell analyses. (**B**) Tumor volumes in B16-OVA inoculated mice (*n* = 10 in each group). (**C**–**H**) Intratumor macrophage surface marker expressions analyzed by flow cytometry. CD80 I, CD86 (**D**), H-2I (**E**), I-A^b^ (**F**), CD163 (**G**) and CD206 (**H**) expressions in CD45+CD11b+F4/80+ (macrophage) populations. Fold expression change of mean fluorescence intensity (MFI) was calculated by following the molecule expression in the target sample vs. control sample (as a base = 1) in flow cytometry analysis. (**I**) RNA expression profile in intratumor macrophages. Intratumor macrophages were isolated from B16-OVA-inoculated mice on day14. Total RNA was isolated from the macrophages and subjected to real-time PCR. M1 and M2 associated mRNA expressions were represented in top and bottom heatmaps, respectively. The mRNA expression was quantified by ∆Ct method. Fold change (FC) of mRNA expression was calculated by following the expression in target sample vs. control sample (as a base = 1). The cumulative data were shown as mean ± standard error (SD) of six samples. Student *t*-test was used to analyze data for significant differences. Values of * *p* < 0.05, ** *p* < 0.01 or *** *p* < 0.001 were regarded as significant.

To investigate how C60 modifies macrophage activity in anti-tumor immunity, we established a murine melanoma model. Since macrophages occupy more than 80% of the total intratumor leukocytes in B16-OVA originated melanoma [21], this model is suitable for investigating macrophage activity in anti-tumor immunity. Wild-type (WT) mice received intragastric (i.g.) administration of saline or HK-C60 every day for 28 days, and B16-OVA cells were inoculated into the mice by subcutaneous (s.c.) injection on their back skin on day 14 of the administration schedule. The tumor volumes were measured on days 7 and 14 (day 14 and 28 in the entire experimental schedule, respectively) after tumor inoculation. At day 28, the mice were sacrificed, and collected samples were used for analyses (Figure 1A). Although tumor volumes were similar between these two groups of mice on day 7, there was a significant decrease in the HK-C60 administered group compared to the control group on day 14 after tumor inoculation (Figure 1B). This effect was also observed in a different tumor model, EO771 murine breast cancer. The tumor volume was significantly decreased by HK-C60 administration compared to saline administration in the mice on day 14 of post tumor inoculation (Figure S2). Interestingly, this suppressed tumor growth was observed only in the HK-C60 administration in the LAB strains that we used in this study (Figure S3). Flow cytometry analysis revealed that M1 phenotype (inflammatory) markers, such as CD80, CD86, MHC class I (H-2K^b^), and MHC class II (I-A^b^), were all upregulated (Figure 1C–F), while M2 phenotype markers, CD163 and CD206, were downregulated in intratumor (IT) macrophages in HK-C60-administered mice as compared to those of controls (Figure 1G,H). Gene expression profiling also supported the results of flow cytometry analysis, showing that M1-associated inflammatory cytokine and inducible nitric oxide synthase (iNOS) mRNA expressions were increased in the IT macrophages of HK-C60-administered mice, while M2 cytokines were all downregulated in their mRNA expressions (Figure 1I). Thus, C60 administration suppresses tumor growth accompanied with macrophage polarization predominantly into an inflammatory character.

### 3.2. C60 Enhances Antigen Presentation Function of Macrophages Activating CD8+ T Cells

**Figure 2:**
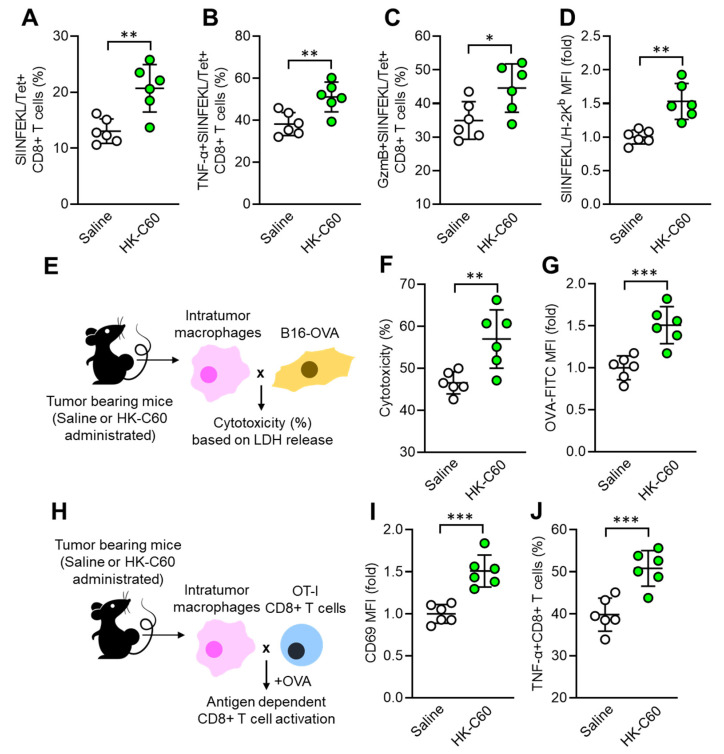
Enhanced antigen presentation activity of IT macrophages in C60-administered mice. Intragastric (i.g.) administration of saline or HK-C60 and B16-OVA inoculation were performed in the mice following protocol represented in Figure 1A. Intratumor leukocytes or macrophages were isolated from each mouse on day 14 of post tumor inoculation and subjected to each analysis. (**A**) Percentage of tumor antigen specific CD8+ T cells analyzed by flow cytometry. (**B**,**C**) Percentages of TNF-α+ (**B**) and Granzyme B+ (**C**) tumor antigen specific CD8+ T cells analyzed by flow cytometry. (**D**) The expression of tumor peptide antigen (SIINFEKL) presenting H-2K^b^ complex in macrophages analyzed by flow cytometry. (**E**) Experimental design of in vitro tumor killing assay. IT macrophages were co-cultured with B16-OVA cells, and the cytotoxicity was measured by LHD release from killed tumor cells. (**F**) Percentage of cytotoxicity of IT macrophages against tumor cells. (**G**) In vitro antigen uptake assay. IT macrophages were cultured with OVA-FITC, then antigen uptake activity was evaluated by intracellularly incorporated OVA following FITC signal analyzed by flow cytometry. (**H**) Experimental design of in vitro antigen presentation assay. IT macrophages were co-cultured with OT-I CD8+ T cells in the presence of OVA protein. The CD8+ T cell activity was analyzed by flow cytometry. (**I**) CD69 expression in CD8+ T cells. (**J**) Percentage of TNF-α+CD8+ T cells. Fold expression change of MFI was calculated by following the molecule expression in the target sample vs. control sample (as a base = 1) in flow cytometry analysis. The cumulative data were shown as mean ± SD of six samples. Student *t*-test was used to analyze data for significant differences. Values of * *p* < 0.05, ** *p* < 0.01 or *** *p* < 0.001 were regarded as significant.

Next, we investigated whether the functionally modified macrophage contributes to substantial anti-tumor immunity in C60 administered mice. Since CD8+ T cells are important players in the cytotoxic effect against tumor [24], we decided to characterize the CD8+ T cell activity as well as antigen presenting function in macrophage. The frequency of tumor antigen (OVA protein-originated SIINFEKL peptide) specific T cell receptor (TCR)-expressing CD8+ T cells was significantly increased in the tumor microenvironment (TME) of HK-C60 administered mice compared with controls (Figure 2A). Additionally, TNF-α and Granzyme B-producing populations were also increased in the tumor antigen-specific CD8+ T cells by HK-C60 administration (Figure 2B,C). In IT macrophages, the expression of tumor antigen presenting MHC class I molecule (SIINFEKL/H-2K^b^ complex) was increased by HK-C60 administration (Figure 2D). In vitro tumor lysis and antigen uptake assays exhibited that IT macrophages in HK-C60 administered mice possessed enhanced cytolytic effect against tumor cells (Figure 2E,F) and OVA-antigen uptake (Figure 2G) compared to control cells, respectively. The enhanced antigen-presenting activity of IT macrophages in HK-C60 administered mice was proven by in vivo antigen presentation assay. The intratumor macrophages of HK-C60 administered mice increased activation marker CD69 expression as well as TNF-α production in OT-I CD8+ T cells in an antigen-dependent manner (Figure 2H–J). Thus, C60 modifies the antigen-presenting activity of IT macrophages resulting in the upregulation of CD8+ T cell activity in the enhanced anti-tumor immunity.

### 3.3. C60 Enhances Mitochondrial Oxidative Metabolism to Increase ATP Production in Macrophages

**Figure 3:**
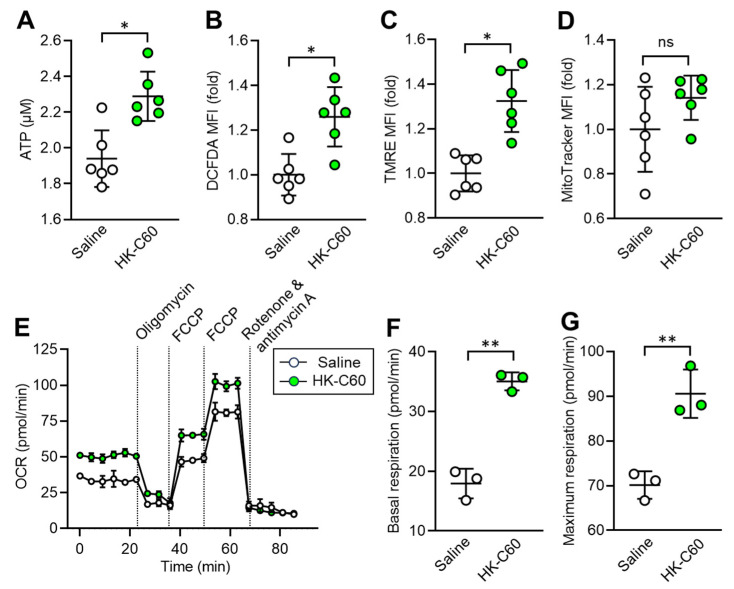
C60 administration enhances mitochondrial activity and ATP production in IT macrophage. Intragastric (i.g.) administration of saline or HK-C60 and B16-OVA inoculation were performed in the mice following protocol represented in Figure 1A. The IT macrophages were isolated from the tumor on day 14 of post tumor inoculation, and the cells were subjected to metabolic analyses. (**A**) ATP concentration measured by luminescence probe. (**B**–**D**) MFIs (fold change) of DCFDA (ROS) (**B**) and TMRE (mitochondrial membrane potential) (**C**) and Mitotracker (mitochondria quantification) (**D**) analyzed by flow cytometry. (**E**–**G**) Real-time metabolic activity assay of IT macrophages using Seahorse analyzer. (**E**) Time dependent OCR, (**F**) Basal respiration, and (**G**) Maximum respiration were represented, respectively. Fold expression change of MFI was calculated by following the molecule expression in the target sample vs. control sample (as a base = 1) in flow cytometry analysis. The cumulative data were shown as mean ± SD of three to six samples. Student *t*-test was used to analyze data for significant differences. Values of * *p* < 0.01 or ** *p* < 0.001 were regarded as significant. ns: not significant.

Since IT macrophage activity was upregulated by HK-C60 administration in the mice, we decided to investigate the metabolism in the cells. It has already been documented that enhanced production of energy source, such as ATP, is directly associated with upregulated inflammatory activity of macrophages [22]. For this purpose, we first measured the ATP level in IT macrophages. Interestingly, intracellular ATP concentration was significantly increased in IT macrophages originating from HK-C60 administered mice compared to that of control cells (Figure 3A). Additionally, ROS production was also increased by HK-C60 administration in IT macrophages (Figure 3B). Following these results, we next characterized mitochondrial activity and numbers in IT macrophages. Mitochondrial membrane potential, which frequently shows an equivalency to oxidative phosphorylation (OXPHOS) level [22,25], was increased in the IT macrophages by HK-C60 administration (Figure 3C), while the number of mitochondria showed no difference in the IT macrophages between saline and HK-C60 administration (Figure 3D). The metabolic activity of IT macrophage was further investigated by using the real-time metabolism analyzer Seahorse. IT macrophages originating from HK-C60 administered mice showed a higher oxygen consumption rate (OCR) than that of control cells, implying that mitochondrial OXPHOS activity was upregulated by HK-C60 administration (Figure 3E). The upregulated mitochondrial metabolic activity was also confirmed by increased basal and maximum respiration in IT macrophages of HK-C60 administered mice (Figure 3F,G). Thus, C60 upregulates macrophage oxidative metabolism, which increases ATP production by enhancing mitochondrial OXPHOS.

### 3.4. C60 Modifies Metabolic Demand as Glycolysis Preferable Manner in Macrophages

**Figure 4:**
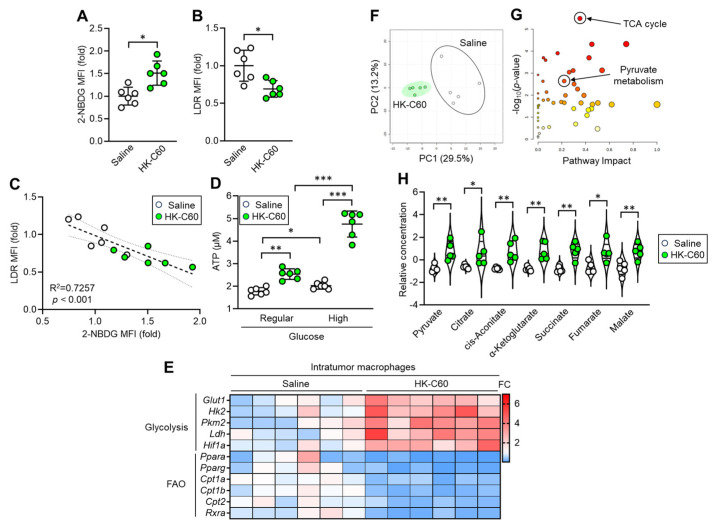
C60 administration upregulates oxidative metabolism allowing increased ATP production in IT macrophages. Intragastric (i.g.) administration of saline or HK-C60 and B16-OVA inoculation were performed in the mice following protocol represented in Figure 1A. IT macrophages were isolated from tumor and used for experiments. (**A**) 2-NBDG MFI (glucose uptake) and (**B**) LDR MFI (lipid uptake) in IT macrophages analyzed by flow cytometry. (**C**) Liner regression assay of 2-NBDG MFI and LDR MFIs. (**D**) In vitro ATP assay in IT macrophage cultured with regular or high concentration of glucose. Fold expression change of MFI was calculated by following the molecule expression in the target sample vs. control sample (as a base = 1) in flow cytometry analysis. (**E**) Gene expression profile of glycolysis and fatty acid oxidation (FAO)-associated genes in IT macrophages analyzed by real-time PCR. The mRNA expression was quantified by *∆*Ct method. FC of mRNA expression was calculated by following the expression in target sample vs. control sample (as a base = 1). (**F**–**H**) Metabolic profile in IT macrophages. Total metabolites were isolated from IT macrophages and subjected to metabolomics. (**F**) PCA analysis. (**G**) Pathway analysis. (**H**) Quantification of metabolites associated with TCA cycle. The cumulative data were shown as mean ± SD of six samples. Student *t*-test or one-way ANOVA was used to analyze data for significant differences. Values of * *p* < 0.05 of ** *p* < 0.01 or *** *p* < 0.001 were regarded as significant. ns: not significant.

Next, we investigated the mechanism by which C60 upregulates oxidative metabolism in IT macrophages. To examine the metabolic preference of the macrophages, we performed in vitro glucose or fatty acid uptake assays using IT macrophages. IT macrophages derived from HK-C60 administered mice showed increased glucose uptake, while fatty acid uptake was decreased compared to those of control cells (Figure 4A,B). Linear regression analysis showed that glucose and fatty acid uptake activities were negatively correlated in the IT macrophages (Figure 4C). In vitro ATP production assay showed that IT macrophages derived from HK-C60 administered mice produced more ATP than control cells in regular glucose medium (fatty acid-free), and it was further increased when the cells were cultured in high glucose condition (Figure 4D). The IT macrophage gene expression profile showed that the expressions of glycolysis-associated genes were upregulated, while fatty acid metabolism-associated genes were downregulated in HK-C60 administered mice compared to controls (Figure 4E). To analyze the details of the metabolic chain, metabolomic analysis was performed in the IT macrophages. Principal component analysis (PCA) showed that the entire metabolomes of IT macrophages were clearly distinct due to HK-C60 administration compared to control treatment (Figure 4F). The pathway analysis revealed that the differences in the identified metabolites were enriched in the tricarboxylic acid (TCA) cycle-associated metabolism between saline and HK-C60 administration. Additionally, the differences were also enriched in the pyruvate metabolic pathway which is tightly associated with TCA cycle driving (Figure 4G). Moreover, pyruvate and intermediate metabolites in the TCA cycle (citrate, cis-aconitate, α-ketoglutarate, succinate, fumarate, and malate) were significantly increased in IT macrophages originated from HK-C60 administered mice compared to control cells (Figure 4H). Thus, C60 enhances aerobic glycolysis rather than fatty acid oxidation for increased ATP production in IT macrophages.

### 3.5. C60 Modifies Macrophage Activity and Metabolism via TLR Signaling in Enhanced Anti-Tumor Immunity

**Figure 5:**
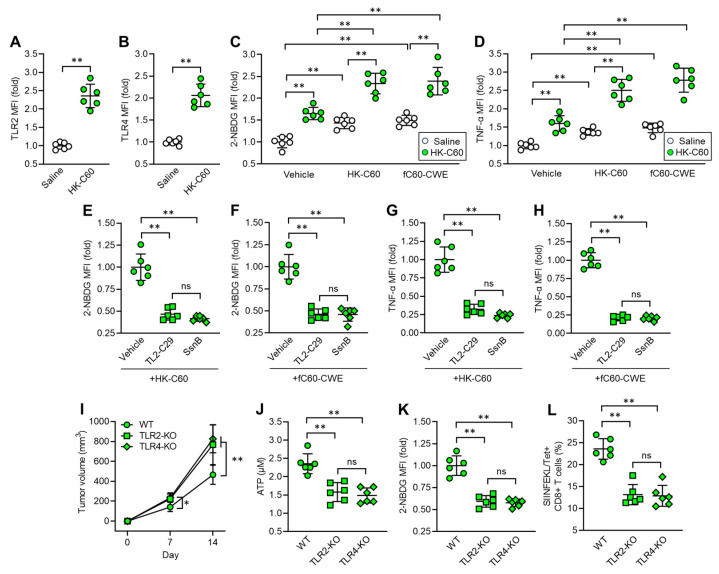
C60 upregulates glycolysis-associated metabolism and immune function via TLR signaling in IT macrophage. Intragastric (i.g.) administration of saline or HK-C60 and B16-OVA inoculation were performed in the mice following protocol represented in Figure 1A. IT macrophages were isolated from tumor and used for experiments. (**A**) TLR2 and (**B**) TLR4 expressions in IT macrophages analyzed by flow cytometry. (**C**) Glucose uptake assay. IT macrophages were cultures with vehicle, HK-C60 or fC60-CWE, and 2-NBDG MFI was analyzed by flow cytometry. (**D**) TNF-α production assay. IT macrophages were cultures with vehicle, HK-C60 or fC60-CWE, and TNF-α MFI was analyzed by flow cytometry. (**E**,**F**) Glucose uptake assay with TLR inhibition. IT macrophages were isolated from HK-C60 supplemented mice, and treated with vehicle, TL2-C29 (TLR2 inhibitor) or SsnB (TLR4 inhibitor). 2-NBDG MFI was measured in the macrophages with HK-60 (**E**) or fC60-CWE (**F**) stimulation by flow cytometry. (**G**,**H**) TNF-α production with TLR inhibition. The IT macrophages were cultured in the same conditions represented in Figure 5E,F. The TNF-α MFI was measured by flow cytometry. (**I**–**K**) Tumor growth in TLR-deficient mice and IT macrophage functional assay. Intragastric (i.g.) administration of HK-C60 and B16-OVA inoculation was performed in the WT, LTR2-KO or TLR4-KO mice following protocol represented in Figure 1A. The tumor volumes were measured after 7 and 14 days of post tumor inoculation. IT macrophages were isolated from tumor and subjected to ATP and glucose uptake assay, and tumor antigen specific CD8+ T cells were also analyzed in the tumor isolated leukocytes. (**I**) Tumor volumes of B16-OVA inoculated mice. *n* = 10 in each group. (**J**) ATP concentration measured by luminescence probe. (**K**) 2-NBDG MFI measured by flow cytometry. (**L**) Percentage of tumor antigen specific CD8+ T cells analyzed by flow cytometry. Fold expression change of MFI was calculated by following the molecule expression in the target sample vs. control sample (as a base = 1) in flow cytometry analysis. The cumulative data were shown as mean ± SD of six samples. Student *t*-test or one-way ANOVA was used to analyze data for significant differences. Values of * *p* < 0.05 or ** *p* < 0.001 were regarded as significant. ns: not significant.

We investigated the mechanism by which C60 induces functional upregulation and metabolic shift towards glycolysis in IT macrophages. In vitro cultures showed that the mRNA expressions of Toll-like receptor (TLR) 2 and TLR4 were upregulated in thioglycolate-elicited peritoneal macrophages (TPMs) upon stimulation with HK-C60 (Figure S4A,B). These receptors played crucial roles in the activation of TPMs, as revealed by inhibition of the signaling, which abolished cellular activation of TPMs on exposing with HK-C60 (Figure S4C). Additionally, TLR2 or TLR4 signaling inhibition decreased glucose uptake in TPMs stimulated with HK-C60 (Figure S4D). Given these findings, we characterized TLR expressions in IT macrophages originating from saline or HK-C60 administered mice. The protein expressions of TLR2 and TLR4 were increased in the IT macrophages originating from HK-C60 administered mice compared to control cells (Figure 5A,B). Since LAB cell wall structures are frequently recognized by TLRs as well as other pattern recognition receptors (PPRs) on immune cells, leading to their functional modification [6,26], we investigated the role of the bacterial signal recognition through TLR2 and TLR4 in functional modification of IT macrophages. For this purpose, we utilized fragmented (f) C60-CWE and compared its effects with those of HK-C60. Even though orally administered HK-C60 is recognized and captured by intestinal myeloid cells, such as DCs and macrophages, and upregulates their functions, these cells are hardly able to migrate into the TME in mice. Therefore, we designed the experiments using fC60-CWE to mimic the possible condition in which intestinally degraded C60 structural components are distributed throughout the entire body and eventually activate IT macrophages. In fact, intraperitoneal (i.p.) injection of fC60-CWE activated spleen and liver macrophages (Figure S5A,B). Moreover, the fC60-CWE i.p. injected mice showed tumor suppression, a similar effect to the observation in HK-C60 i.g. administration in the B16-OVA-inoculated mice (Figure S5C). These results proved that degraded C60 structural substances are sensed by peripheral macrophages and induce their functional modifications. In vivo culture showed that HK-C60 or fC60-CWE stimulation increased glucose uptake and TNF-α production in IT macrophages originating from HK-C60 administered mice more than the cells isolated from control mice (Figure 5C,D). However, the stimulatory effect of HK-C60 or fC60-CWE was abolished by inhibition of TLR-signaling in IT macrophages. The signal inhibition for TLR2 (TL2-C29 treatment) or TLR4 (SsnB treatment) suppressed the upregulation of glucose uptake as well as TNF-α production induced by the stimuli in IT macrophages isolated from HK-C60 supplemented mice (Figure 5E–H). To investigate the contribution of TLRs to the enhanced anti-tumor activity of macrophages in HK-C60 administration, we established a melanoma model with HK-C60 administration in WT, TLR2 or TLR4-KO mice following the protocol represented in Figure 1A. Interestingly, either TLR2-KO or TLR4-KO mice showed progressed tumor growth compared to WT mice in HK-C60 administered condition (Figure 5I). The ATP level and glucose uptake were significantly decreased in TLR2 or TLR4-deficiency in IT macrophages as compared to those of WT cells (Figure 5J,K). Additionally, the frequencies of tumor antigen-specific CD8+ T cells were decreased in the TME of TLR2-KO or TLR4-KO mice compared to that of WT mice (Figure 5L). Thus, TLR signaling plays a crucial role in the activation and shifting into glycolysis pre-dominant metabolism in IT macrophages in C60 administration.

## 4. Discussion

This study revealed that C60 modifies metabolic preference in macrophages, predominantly shifting towards glycolysis in increased ATP production, which is one of the key features in enhancing their function and anti-tumor immunity. This is the first report providing evidence that probiotic LAB strengthen macrophage function enabling them to fight against tumors more effectively than under natural conditions. Additionally, it is a new finding that probiotic LAB alter metabolic preference in macrophages resulting in an increased ATP level. Thus, both our previous and current studies [19], even though each study targeted different cell types, provide strong evidence that C60 is a probiotic strain with a function in enhancing anti-tumor immunity. This function signifies a new adaptation of this strain, expecting functional modifications in the host’s innate–adaptive immune axis that eventually strengthen the CD8+ T cell-based anti-tumor effect.

Although the anti-tumor phenotype observed in C60 administration was obvious in the mouse model of this study, we have not yet obtained a clear answer to a universal question in probiotics; how LAB effects are transferred and distributed from the gut to distal tissues/organs. It has been a well-documented phenomenon that LAB administration modifies immune responses not only in the gut but also systemically [27,28]. However, no report provides an answer to the detailed mechanism. We have proposed two hypotheses as possibilities in the global functional modification in immunity: one is LAB structural components mediated manner and the second is their byproducts mediated manner. For the first possibility, the cleaved or digested LAB structural components can be distributed in the body and trigger functional modifications in immune cells in peripheral tissues and organs. There is no doubt that orally administered LAB should be initially recognized by gut immune cells, mostly epithelial and lamina propria DCs, as reported [29,30]. These cells capture LAB and receive stimulatory signals by themselves for functional upregulation, while the LAB might be digested in the cytosol in the meantime [7]. For instance, the LAB cell wall structure composed of various glycans and proteins can be degraded into tiny fragments that could travel throughout the entire body via the blood stream [31,32]. Eventually, these components can prime the immune cells leading to enhanced immune activity. In fact, our results suggested that fragmented C60-CWE enhanced macrophage function and metabolic activity, which was at a level close to the cells exposed to whole HK-C60. This in vitro experiment might mimic the in vivo macrophage response in C60-administered mice. In this study, we focused on the degraded structural component of C60 and tried to explain the distal effect in IT macrophage functional upregulation; however, our observation was insufficient to explain the hypothesis. To prove this concept, we need to conduct an experiment using ^13^C-labeled C60 and trace the radioisotope signal in peripheral immune cells. Additionally, it is also possible that C60 stimulatory signals alter the bone marrow (BM) environment in mice, and this change leads to the continuous production of functionally modified myeloid cells. Similar to the concept of immunological memory function in the lymphoid system, BM myeloid progenitors are influenced by peripheral environment changes, which are transmitted to the BM, and future mature myeloid cells will gain modified function when they are differentiated [33]. As a second possibility, we must consider influences of LAB-derived metabolites and peptides shaping distal immune functional enhancement. It has already been documented that probiotic LAB produce these bioactive factors which directly affect cellular functions [34,35]. There is a possibility that C60 produces immuno-activating metabolites and/or peptides which contribute to enhancing anti-tumor activity of macrophages.

While studies have been widely conducted, probiotic LAB still have unknown effects on immunity. As we are presenting a novel LAB effect in cancer, some LAB strains may possess protective effects against specific diseases that offer far greater advantages than the traditional understanding of LAB contributions to our health. To create future probiotic LAB studies that are more translational than the current trend, we may better adapt LAB into various models that have not yet been tried in the field. Additionally, investigations must be performed by focusing on unlimited biological phenomena. Traditional cytokine production and cellular activation focusing on immune cells are not sufficient approaches to reveal novel LAB functions. Likewise, in our current study employing metabolic assays, a different angle must be used in future probiotic LAB studies.

## 5. Conclusions

*Lactococcus lactis* subsp. *cremoris* C60 stimulates macrophages via TLR2 and TLR4, inducing an inflammatory phenotype and shifting their metabolic preference to glycolysis in the TME. This metabolic change increases ATP production, supporting energy demand sufficiently for their functional enhancement and resulting in anti-tumor CD8+ T cell activation in a murine melanoma model.

## Data Availability

The original data presented in the study are represented in the Supplementary Material. Further inquiries can be directed to the corresponding authors.

## Supplementary Materials

The following supporting information can be downloaded at: https://www.mdpi.com/article/10.3390/cancers16101928/s1, Figure S1: Tumor growth in murine melanoma model at different doses of HK-C60; Figure S2: Tumor growth in murine breast cancer model; Figure S3: C60 specifically suppresses tumor growth in murine melanoma; Figure S4: TLR-dependent cellular activation and upregulation of glucose uptake in TPMs; Figure S5: Fragmented C60-CWE has the same effect as HK-C60 in macrophage functional modification; Table S1: Antibody list for flow cytometry; Table S2: Primer sequences for real-time PCR.
