# Supplementary material for: Lactococcus lactis subsp. cremoris C60 Upregulates Macrophage Function by Modifying Metabolic Preference in Enhanced Anti-Tumor Immunity
Source: Cancers (Basel). 2024 May 18;16(10):1928. doi: 10.3390/cancers16101928 (PMC11120145; doi:10.3390/cancers16101928)
Supplement: Supplementary file 1 [file cancers-16-01928-s001.zip › cancers-2996306-supplementary.pdf]

Lactococcus lactis subsp. _cremoris_ C60 upregulates macrophage function by modifying metabolic preference in enhanced anti-tumor immunity

Suguru Saito*, Duo-Yao Cao, Toshio Maekawa, Noriko M. Tsuji and Alato Okuno*

*Correspondence: Suguru Saito, Ph.D., E-mail: Suguru.Saito@cshs.org, Tel: +1-213-823-0661; Alato Okuno, Ph.D., E-mail: a-okuno@shibata.ac.jp

Supplementary Materials

Supplementary Protocol

Thioglycolate-elicited peritoneal macrophages (TPMs) preparation

The mice received an intraperitoneal (i.p.) injection of 3 mL of thioglycolate and the infiltrated leukocytes were harvested from the peritoneum at day 4 (between 84 to 96 h) post injection. The cells were seeded into 100 mm cell culture dishes in RPMI complete medium (RPMI1640 supplemented with 10% of FBS, 100 U/mL of penicillin and 100 μg/mL of streptomycin) and incubated at 37°C for 3 h. The adherent cells, which are enriched TPMs, were washed with PBS three times and then detached by gentle scraping in PBS/2% FBS. The purity of TPMs was assessed by flow cytometry. The samples with more than 90% of CD11b+F4/80+ population were used for subsequent experiments.

Fragmented C60-CWE injection and tumor inoculation

The mice received i.p. injection of saline (200 μL) or fragmented (f)C60-CWE (200 μL of suspension derived from 1.0×10^9 CFU/mL in saline) every 3 day for 28 days. In day 14 of i.p. injection, the mice received tumor inoculation (1.0×10^6 of B16-OVA cells in 100 μL of PBS) on the back skin by subcutaneous (s.c.) injection. Tumor volume was measured at day 7 and day 14 after tumor inoculation in each mouse. For some experiments, macrophages were isolated from spleen or liver using EasySep™ Mouse F4/80 Positive Selection Kit (Stemcell Technologies) in the mice received i.p. injection for 14 days (non-tumor). The purity of macrophages was assessed by flow cytometry, and samples with more than 90% of CD11b+F4/80+ population were used for experiments. The isolated macrophages were subjected to *in vitro* stimulation assay. The macrophages (2.5×10^6/mL) were cultured with vehicle (PBS) or LPS (1 μg/mL) at 37°C for 6 h, then CD86 expression was analyzed by flow cytometry.

Supplementary Table

Table S1. Antibody list for flow cytometry

Antigen	Fluorochrome	Clone	Company
CD3	FITC	17A2	BioLegend
CD4	APC	GK1.5	BioLegend
CD8	Pacific Blue	53-6.7	BioLegend
CD11b	PE-Cy7	M1/70	BioLegend
CD45	PerCP-Cy5.5, PE-Cy7	30-F11	BioLegend
CD69	PE	H1.2F3	BioLegend
CD80	FITC	16-10A1	BioLegend
CD86	APC	GL-1	BioLegend
CD163	APC-Cy7	S15049F	BioLegend
CD206	Brilliant Violet 711	C068C2	BioLegend
F4/80	Brilliant Violet 650	BM8	BioLegend
I-A^b	FITC	25-9-17	BioLegend
H-2K^b	Pacific Blue	AF6-88.5	BioLegend
TNF-α	APC, PerCP-Cy5.5	MP6-XT22	BioLegend
Granzyme B	PerCP-Cy5.5	QA16A02	BioLegend
SIINFEKL/H-2K^b	PE, APC	25-D1.16	BioLegend
SIINFEKL/H-2K^b Tetramer	PE	-	MBL

Table S2. Primer sequences for real-time PCR

Target gene	Forward (5'-3')	Reverse (5'-3')
Il1b	GCAACTGTTCCTGAACTCAACT	ATCTTTTGGGGTCCGTCAACT
Il6	CCAAGAGGTGAGTGCTTCCC	CTGTTGTTCAGACTCTCTCCCT
Il12a	CCCTTGCCCTCCTAAACCAC	AAGGAACCCTTAGAGTGCTTACT
Il12b	TGGTTTGCCATCGTTTTGCTG	ACAGGTGAGGTTCACTGTTTCT
Tnf	GACGTGGAACTGGCAGAAGAG	TTGGTGGTTTGTGAGTGTGAG
Nos2	GTTCTCAGCCCAACAATACAAGA	GTGGACGGGTCGATGTCAC
Il4	GGTCTCAACCCCCAGCTAGT	GCCGATGATCTCTCTCAAGTGAT
Il10	GCTCTTACTGACTGGCATGAG	CGCAGCTCTAGGAGCATGTG
Il13	CCTGGCTCTTGCTTGCCTT	GGTCTTGTGTGATGTTGCTCA
Arg1	CTCCAAGCCAAAGTCCTTAGAG	AGGAGCTGTCATTAGGGACATC
Glut1	CAGTTCGGCTATAACACTGGTG	GCCCCCGACAGAGAAGATG
Hk2	TGATCGCCTGCTTATTCACGG	AACCGCCTAGAAATCTCCAGA
Pkm2	GCCGCCTGGACATTGACTC	CCATGAGAGAAATTCAGCCGAG
Ldh1	TGTCTCCAGCAAAGACTACTGT	GACTGTACTTGACAATGTTGGGA
Hif1a	ACCTTCATCGGAAACTCCAAAG	ACTGTTAGGCTCAGGTGAACT
Ppara	AGAGCCCCATCTGTCCTCTC	ACTGGTAGTCTGCAAAACCAAA
Pparg	TCGCTGATGCACTGCCTATG	GAGAGGTCCACAGAGCTGATT
Cpt1a	CTCCGCCTGAGCCATGAAG	CACCAGTGATGATGCCATTCT
Cpt1b	GCACACCAGGCAGTAGCTTT	CAGGAGTTGATTCCAGACAGGTA
Cpt2	CAGCACAGCATCGTACCCA	TCCCAATGCCGTTCTCAAAAT
Rxra	ATGGACACCAAACATTTCCTGC	CCAGTGGAGAGCCGATTCC
Gapdh	AGGTCGGTGTGAACGGATTTG	TGTAGACCATGTAGTTGAGGTCA

Supplementary Figure

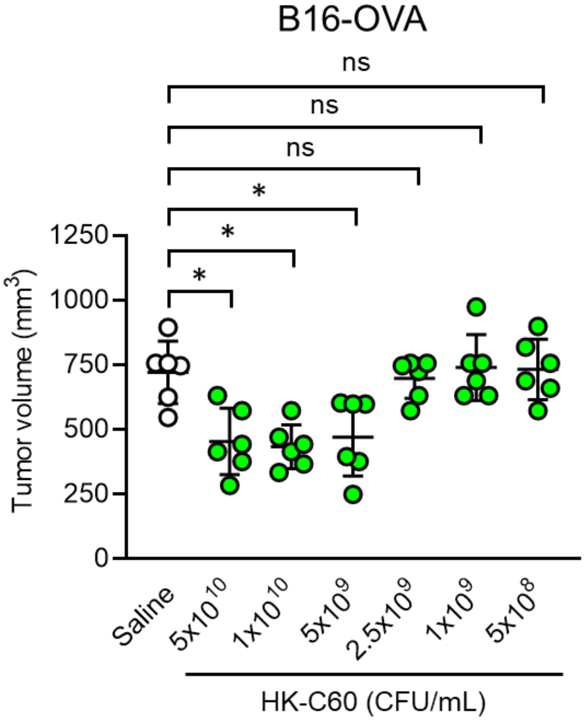

Figure S1. Tumor growth in murine melanoma model at different does of HK-C60. Oral administration and tumor inoculation was performed by following protocol represented in Figure 1A. The mice first received oral administration of saline, HK-C60 (indicated CFU/mL) for 14 days, and B16-OVA cells were inoculated by s.c. injection into back skin (n=6 in each group). The tumor volumes were measured after 14 days of tumor inoculation (in day 28 of oral administration). The cumulative data were shown as mean ± SD. One-way ANOVA was used to analyze data for significant differences. Values of **p* < 0.01 was regarded as significant.

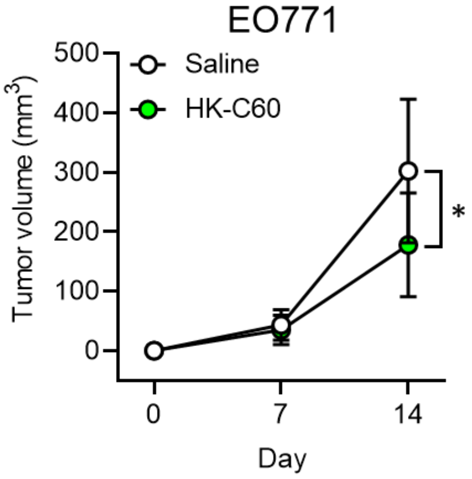

Figure S2. Tumor growth in murine breast cancer model. Oral administration and tumor inoculation was performed by following protocol represented in Figure 1A. The murine breast cancer model was established by intramammary (i.m.) injection of EO771 cells ($2.0x10^6$ cells/100 uL of PBS) into the mice (*n*=10 in each group). The tumor volumes were measured is 7 and 14 days of tumor inoculation (in day 21 and 28 of oral administration). The cumulative data were shown as mean ± standard error (SD). Student *t*-test was used to analyze data for significant differences. Values of **p* < 0.05 was regarded as significant.

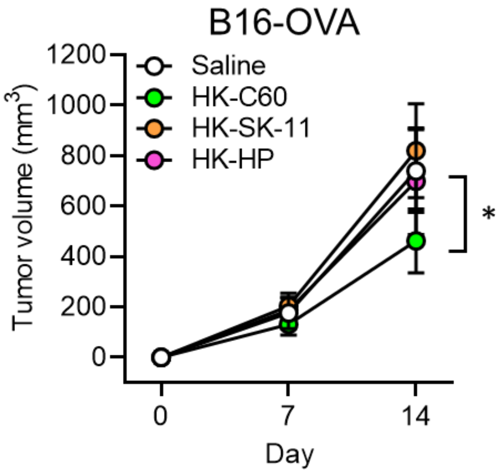

Figure S3. C60 specifically suppresses tumor growth in murine melanoma. The murine melanoma model was established by B16-OVA inoculation following the protocol represented in Figure 1A. The mice first received oral administration of saline, HK-C60, HK-SK-11 or HK-HP for 14 days, and B16-OVA cells were inoculated by s.c. injection into back skin (n=10 in each group). The tumor volumes were measured in 7 and 14 days of tumor inoculation (in day 21 and 28 of oral administration). The cumulative data were shown as mean ± SD. Student t-test was used to analyze data for significant differences. Values of $*p < 0.01$ was regarded as significant.

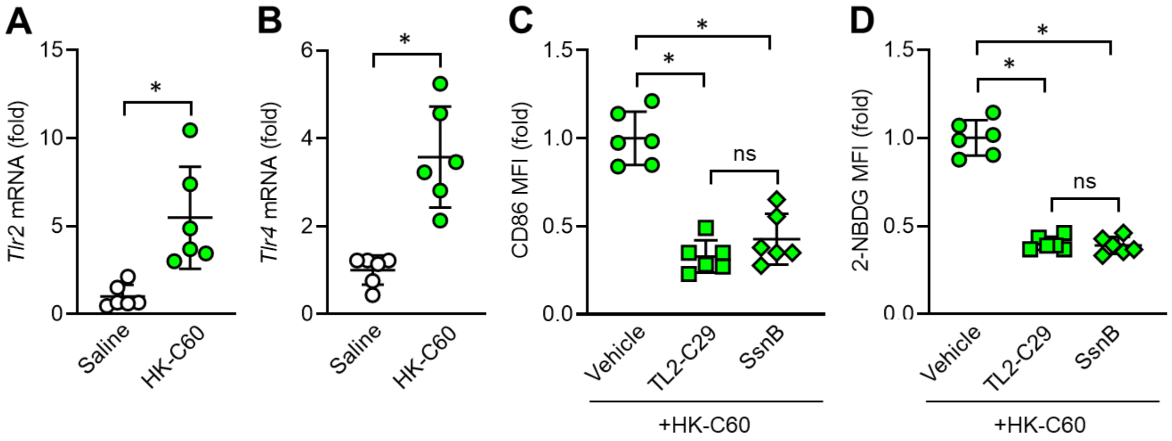

Figure S4. TLR-dependent cellular activation and upregulation of glucose uptake in TPMs. A-B) Gene expressions of TLRs in TPMs. TPMs were cultured with vehicle (PBS) or HK-C60, then mRNA expressions of TLR2 (A) and TLR4 (B) were quantified by real-time PCR. Fold change of mRNA expression was calculated by following the expression in target sample vs control sample (as a base=1). C) CD86 expression of TPMs. TPMs were pre-treated with vehicle (DMSO), TL2-C29 or SsnB following HK-SA stimulation. The CD86 expressions were analyzed by flow cytometry. D) Glucose uptake in TPMs. TPMs were pre-treated with the same condition described in (C) followed by glucose uptake activities were analyzed by flow cytometry. Fold expression change of mean fluorescence intensity (MFI) was calculated by following the molecule expression in the target sample vs control sample (as a base=1) in flow cytometry analysis. The cumulative data were shown as mean ± SD of six samples. Student t-test or one-way ANOVA was used to analyze data for significant differences. Values of $*p < 0.01$ was regarded as significant. ns: not significant.

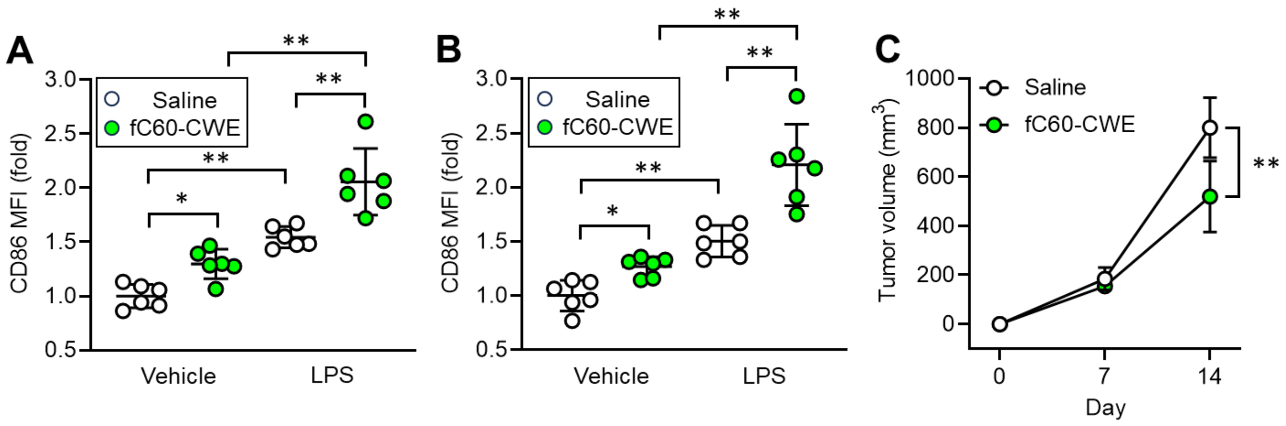

Figure S5. Fragmented C60-CWE has the same effect to HK-C60 in macrophage functional modification. A-B) *In vitro* macrophage stimulation assay. Macrophages were isolated from spleen of liver of the mice received i.p. injection of saline or fC60-CWE every day for 14 days. The macrophages were cultured with vehicle (PBS) or LPS, then cell activation statuses were analyzed by flow cytometry. A) CD86 expression of spleen macrophages. B) CD86 expression of liver macrophages. C) B16-OVA-derived tumor growth. The mice received i.p. injection of saline or fC60-CWE every day for 28 days. At day 14 of i.p. injection, B16-OVA cells were inoculated to their back skin by s.c. injection. The tumor volumes were measured at day 7 and 14 of post tumor inoculation. (n=10 in each group). The cumulative data were shown as mean ± SD of six or ten samples. Student t-test or one-way ANOVA was used to analyze data for significant differences. Values of *$p < 0.01$ or **$p < 0.001$ was regarded as significant.
